# Strong population genetic structuring in an annual fish, *Nothobranchius furzeri*, suggests multiple savannah refugia in southern Mozambique

**DOI:** 10.1186/1471-2148-13-196

**Published:** 2013-09-12

**Authors:** Veronika Bartáková, Martin Reichard, Karel Janko, Matej Polačik, Radim Blažek, Kathrin Reichwald, Alessandro Cellerino, Josef Bryja

**Affiliations:** 1 Institute of Vertebrate Biology, Academy of Sciences of the Czech Republic, Květná 8, Brno 603 65, Czech Republic; 2 Department of Botany and Zoology, Faculty of Science, Masaryk University, Kotlářská 2, Brno 611 37, Czech Republic; 3 Institute of Animal Physiology and Genetics, Academy of Sciences of the Czech Republic, Veveří 97, Brno 602 00, Czech Republic; 4 Leibniz Institute for Age Research – Fritz Lipmann Institute, Beutenbergstrasse 11, Jena 07745, Germany; 5 Scuola Normale Superiore, Plazza dei Cavalieri 6, Pisa 56100, Italy

**Keywords:** Temporary pool, Phylogeography, Population genetics, Cyprinodontiformes, Senescence, Pluvials, Pleistocene climate changes, Dispersal, Founder effect, Killifish

## Abstract

**Background:**

Intraspecific genetic variation of African fauna has been significantly affected by pronounced climatic fluctuations in Plio-Pleistocene, but, with the exception of large mammals, very limited empirical data on diversity of natural populations are available for savanna-dwelling animals. *Nothobranchius furzeri* is an annual fish from south-eastern Africa, inhabiting discrete temporary savannah pools outside main river alluvia. Their dispersal is limited and population processes affecting its genetic structure are likely a combination of those affecting terrestrial and aquatic taxa. *N. furzeri* is a model taxon in ageing research and several populations of known geographical origin are used in laboratory studies. Here, we analysed the genetic structure, diversity, historical demography and temporal patterns of divergence in natural populations of *N. furzeri* across its entire distribution range.

**Results:**

Genetic structure and historical demography of *N. furzeri* were analysed using a combination of mitochondrial (partial cytochrome *b* sequences, 687 bp) and nuclear (13 microsatellites) markers in 693 fish from 36 populations. Genetic markers consistently demonstrated strong population structuring and suggested two main genetic groups associated with river basins. The split was dated to the Pliocene (>2 Mya). The northern group inhabits savannah pools across the basin of the intermittent river Chefu in south-western Mozambique and eastern Zimbabwe. The southern group (from southernmost Mozambique) is subdivided, with the River Limpopo forming a barrier (maximum divergence time 1 Mya). A strong habitat fragmentation (isolated temporary pools) is reflected in significant genetic structuring even between adjacent pools, with a major influence of genetic drift and significant isolation-by-distance. Analysis of historical demography revealed that the expansion of both groups is ongoing, supported by frequent founder effects in marginal parts of the range and evidence of secondary contact between Chefu and Limpopo populations.

**Conclusions:**

We demonstrated: (1) ancient (pre-Pleistocene) divergence between the two main *N. furzeri* lineages, their recent secondary contact and lack of reproductive isolation; (2) important genetic structuring attributed to the fragmented nature of their environment and isolation-by-distance, suggesting that dispersal is limited, occurs over short distances and is not directly associated with river routes; (3) an apparent role of the River Limpopo as a barrier to dispersal and gene flow.

## Background

Phylogeography and historical demography play crucial roles in explaining species distributions and are instrumental in revealing episodes of fragmentation and subsequent expansions connected to environmental and climatic fluctuations [1,2]. Most phylogeographic research has been focused on the Northern Hemisphere, often linked to the Pleistocene glacial cycles. Our knowledge of biogeographical patterns and processes in the Southern Hemisphere is relatively poor [3], and it is especially marked in non-forest biomes. In sub-Saharan Africa studies on large mammals living in savannah have identified large-scale phylogeographic patterns and provided a basic understanding of the major biogeographic regions at a continental scale [4], though studies with small-scale resolution are still rare [5-8].

In Africa, Pleistocene climatic fluctuations have resulted in more arid conditions during glacial periods, as large masses of water were deposited in continental glaciers, alternating with more mesic conditions during interglacials (termed pluvials in the tropics). Thus African ecosystems have undergone a series of aridification events, accompanied by spreading savannah habitats, since the Pliocene, with relatively wetter periods between 2.7-2.5 Mya, 1.9-1.7 Mya and 1.1-0.9 Mya [9]. Further aridification is documented in the late Pleistocene and, notably, the last interpluvial (130–114 kya) was coincident with a series of extreme droughts in eastern Africa [10]. Studies on large savannah-dwelling mammals demonstrated their less pronounced phylogeographic structure and high level of genetic variation in Southern Africa, suggesting a long-standing persistence of large savannahs in this part of the continent (reviewed in [4]). However, research from temperate areas in the northern latitudes led to understanding that large and highly mobile animals are not ideal taxa to specify the location and character of past refugia (e.g. [11]). On the contrary, species with very low dispersal ability and strong dependence on particular habitat have provided much more detailed picture of phylogeographical patterns and processes, for example the identification of unexpected cryptic refugia (e.g. [12]) or “refugia within refugia” (e.g. [8,13]).

Killifishes (Cyprinodontiformes) are an ancient fish clade with a predominantly Gondwanan distribution and high species richness in Africa [14]. African killifish (family Nothobranchiidae) include several clades that show radiations in specific habitat conditions, such as small rain forest streams, margins of large rivers or brackish zones [15]. A notable clade, comprising the genus *Nothobranchius* (59 species; [16]) from East Africa and related monotypic genera *Pronothobranchius* and *Fundulosoma* from West Africa, has adapted to a unique environment of temporary savannah pools [15]. The pools inhabited by *Nothobranchius* are completely separated from river systems and typically not inhabited by other fishes except lungfish (*Protopterus* spp.). The pools are strictly seasonal and filled with rainwater at the beginning of the rainy season. Pools desiccate during the dry season, with the duration of pools depending on local climate and ranging from 3–11 months [15,17,18]. In some years, a major flooding may connect several pools via flooded savannah and may also connect pools with intermittent streams, though such events appear to be rare. Importantly, specific soil conditions (the presence of alluvial vertisols) are necessary for the occurrence of *Nothobranchius *[17,19], limiting dispersal and occurrence of *Nothobranchius* across the savannah to specific habitat patches. Therefore, population processes affecting genetic structure of *Nothobranchius* are predicted to be a combination of processes that are unique to *Nothobranchius* and these affecting terrestrial and aquatic taxa.

All *Nothobranchius* species are annual. Fish hatch soon after a pool fills with water [15,20], grow rapidly, achieve sexual maturity within a few weeks [21], and reproduce daily thereafter [22]. Adult lifespan is limited by habitat desiccation, with the following generation surviving in the form of diapaused embryos in desiccation-resistant egg envelopes encased in dry mud [19]. Populations of *Nothobranchius* seldom co-occur with other teleost fish species [23]. Dispersal by *Nothobranchius* may be limited to occasional large-scale floods in years with unusually high rainfall, which may transport live fish among savannah pools. Alternatively, eggs encased in mud may be carried attached to the bodies of large herbivores, as reported for aquatic invertebrates and macrophytes [24], or possibly by waterbirds.

Here we use *Nothobranchius furzeri* Jubb to investigate phylogeographic patterns and demographic processes in this group of annual, savannah-adapted fish. Its rapid development, short lifespan and age-dependent deterioration of physiological functions have made it a valuable vertebrate model organism in ageing research [25]. In contrast to that, little is known about natural populations [19]. *Nothobranchius furzeri* is distributed in southern Mozambique [19], with a single locality known in Zimbabwe (Sazale Pan at Gona Re Zhou National Park, Chefu basin, close to the Mozambican border) [26]. Within its geographical range this species is restricted to pools associated with vertisol soils, while it is absent from pools associated with sandy and lateritic soils [17,19]. Previous research on the phylogenetic relationships of Mozambican *Nothobranchius* included samples from several *N. furzeri* populations [27]. The mitochondrial marker *COI* (cytochrome oxidase I) suggested high spatial structuring within the range of all species investigated, but the three nuclear sequences used in the study (*GHITM*, *Cx32.2*, *PNP*) had insufficient power to resolve intraspecific relationships.

Here, we provide the first detailed study of the phylogeographic patterns and demographic processes in *N. furzeri*, covering the entire distribution range of the species using a combination of mitochondrial gene *CYTB* (cytochrome *b*) and 13 nuclear microsatellite markers. Specifically, we tested following hypotheses: (1) *N. furzeri* is composed of three reproductively isolated clades (sensu [27]), with no secondary contact among them. (2) The main divergence was influenced by Plio-Pleistocene climate changes with allopatric diversification due to savannah fragmentation during pluvials. (3) The populations of savannah-dwelling *Nothobranchius* fishes have expanded during last interpluvial, together with their preferred habitat. (4) Genetic diversity will be lower and genetic structure higher on the range periphery, where populations are exposed to more frequent extinction-recolonization processes associated with strong genetic drift.

## Methods

### Sampling

**Figure 1:**
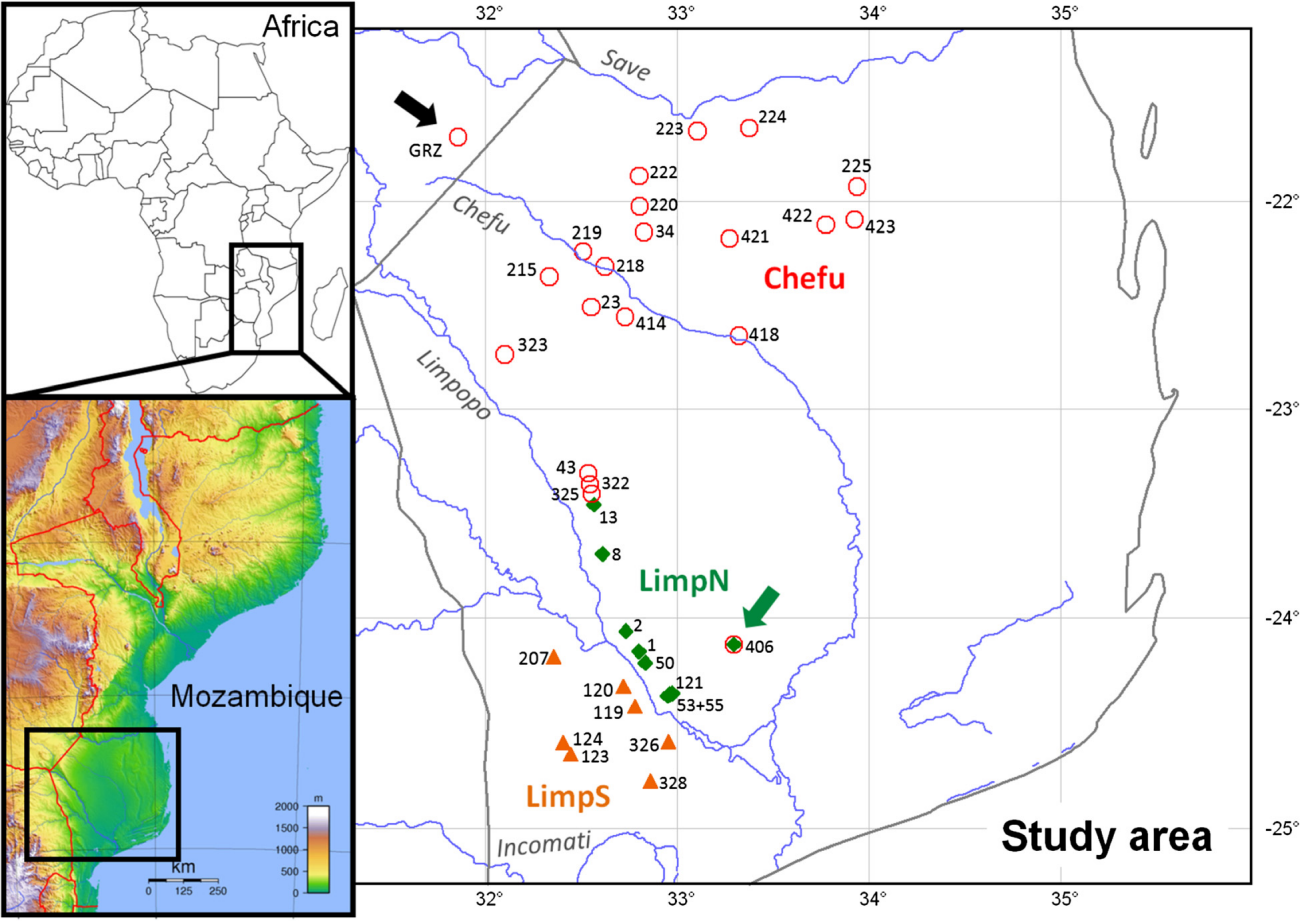
**Map showing the distribution of 36 sample sites.** Different symbols indicate the distribution of three main mtDNA haplogroups (called Chefu, LimpN, and LimpS). Numbers of localities correspond to Table 1. Haplotypes from the two haplogroups (Chefu and LimpN) were found in Pop406 (marked by the green arrow). Note that LimpS includes the localities from both the Limpopo and Incomati basins. Black arrow shows the position of the locality at Gona Re Zhou National Park in Zimbabwe (GRZ) that was excluded from some analyses because the individuals originated from a captive population. The inset figures show the position of the study area within Africa and Mozambique.

Fish were collected during 8 field trips between 2008 and 2012, visiting 336 individual savannah pools across southern and central Mozambique, with the aim of sampling pools across the entire savannah habitat in that region. From a total of 163 pools populated by *Nothobranchius* spp., 73 pools were inhabited by *N. furzeri*, all of them south of the River Save (Figure 1). Fish were collected using dip and Seine nets. Species identification took place in the field, and at most sites a small fin clip from the caudal fin was taken and stored in 96% ethanol. Only adult fish, clearly identified to species, were sampled. Each population sample consisted of fish collected the same day. Different populations (within and across geographical regions), however, were collected across study years (Table 1). There was no major flood between 2008 and 2012 that would cause a dispersal of fish or eggs across populations. A subsample of captured fish was stored in 5% formaldehyde or 80% ethanol for further reference and phenotype analyses while most fish were released back into the pool. Two GRZ (type locality) specimens were obtained from the breeding stock in Leibniz Institute for Age Research, Jena, Germany. For genetic analysis, *N. furzeri* populations from 36 pools (= localities) were selected which cover the entire known species range (Table 1, Figure 1). Adjacent savannah pools were generally omitted, except for two regions where analyses focussed on small-scale structuring. All fieldwork complied with legal regulations of Mozambique (collection permit 154/II/2009/DARPPE and sample export permit 049MP00518-A/09 of the Mozambican Ministry of Fisheries).

**Table 1:** Summary of analyzed material (31 localities for microsatellites and 36 for mtDNA)

***Pop ID***	***Microsatellites***		***MtDNA***		***Lat***	***Long***	***Alt***	***Year***
	***N***	***IBD***	***N***	***Demo***				
1	20	LimpN	3	LimpN	−24.16	32.80	59	2010
2	24	LimpN	2	LimpN	−24.06	32.73	77	2008
8	21	LimpN	3	LimpN	−23.69	32.61	118	2008
13	25	LimpN	3	LimpN	−23.46	32.56	129	2008
23	21	Chefu	3	Chefu	−22.51	32.55	128	2008
34	22	Chefu	3	Chefu	−22.15	32.82	124	2010
43	21	Chefu	7	Chefu	−23.31	32.53	91	2008
50	25	LimpN	3	LimpN	−24.22	32.83	49	2008
53	26	LimpN	3	LimpN	−24.37	32.95	34	2008
55	28	LimpN	2	LimpN	−24.36	32.96	33	2008
119	23	LimpS	7	LimpS	−24.42	32.78	48	2010
120	34	LimpS	5	LimpS	−24.33	32.72	56	2008
121	36	LimpN	3	LimpN	−24.36	32.97	30	2008
123	-	-	4	LimpS	−24.65	32.44	88	2008
124	24	LimpS	6	LimpS (4)	−24.59	32.40	96	2008
207	20	LimpS	6	LimpS	−24.19	32.36	136	2009
215	20	Chefu	2	Chefu	−22.36	32.33	181	2009
218	-	-	5	Chefu	−22.31	32.62	149	2009
219	20	Chefu	3	Chefu	−22.24	32.51	176	2009
220	13	Chefu	3	Chefu	−22.02	32.80	146	2009
222	22	Chefu	3	Chefu	−21.87	32.80	158	2009
223	18	Chefu	5	Chefu	−21.66	33.10	149	2009
224	-	-	4	Chefu	−21.64	33.37	130	2009
225	11	Chefu	5	Chefu	−21.93	33.94	146	2009
322	23	Chefu	5	Chefu	−23.36	32.54	142	2010
323	20	Chefu	7	Chefu	−22.74	32.10	220	2010
325	-	-	6	-	−23.41	32.55	134	2010
326	24	LimpS	5	LimpS	−24.59	32.95	30	2010
328	-	-	1	-	−24.45	32.86	26	2010
406	20	-	6	-	−24.13	33.29	34	2011
414	20	Chefu	6	Chefu	−22.55	32.73	97	2011
418	23	Chefu	5	Chefu	−22.65	33.32	68	2011
421	21	Chefu	5	Chefu	−22.18	33.27	105	2011
422	24	Chefu	3	Chefu	−22.11	33.77	156	2011
423	22	Chefu	6	Chefu (5)	−22.09	33.92	120	2011
GRZ	2	-	1	-	−21.69	31.86	422	2008
Total	673		149	132				

*IBD* group of populations for which isolation-by-distance was analysed, *Demo* group of populations used for analyses of historical demography (number of individual sequences used for analysis of historical demography is shown in parentheses if different from *N*), latitude (*Lat*), longitude (*Long*), altitude (*Alt*) and year of tissue collection (*Year*) of locations of particular populations (*Pop ID*). The populations 123, 124, and 328 geographically belong to the Incomati basin; however this group has never been identified as separate unit.

### Genotyping of microsatellites and mtDNA

In total, 673 individuals collected at 31 localities (Table 1) were genotyped at 13 microsatellite loci in four multiplex PCR sets. PCR products were separated on the ABI Prism® 3130 Genetic Analyzer (Applied Biosystems) and analysed using GeneMapper® v. 3.7 (Applied Biosystems) (see Additional file 1 for more details). Partial mitochondrial gene *CYTB* was amplified using primers FW40 (GCA AAT GAC TCC CTA ATT GAC C) and REV1019 (CCT CCA ATT CAT GTT AGG GTG) designed on the basis of the mtDNA sequence of *N. furzeri* (GenBank Accession NC_011814). PCR products were sequenced directly using BigDye Terminators v. 3.1 chemistry (Applied Biosystems) (see Additional file 1 for more details).

### Analysis of population genetic structure in the distribution range of *N. furzeri*

• Network analysis of mtDNA variation

Sequence variation in *CYTB* (687 bp fragment in 149 individuals from 36 populations; Table 1) was visualised as a haplotype network using the median-joining algorithm in Network 4.610 [28]. All sequences were geo-referenced and the distribution of the main genetic groups was plotted onto map using PanMap software (http://www.pangaea.de/software/PanMap).

• *F*_ST_ and STRUCTURE - microsatellite data

We estimated the proportion of null alleles (*NA*) at each locus and population in FREENA [29]. Linkage disequilibrium among 13 polymorphic loci was examined in each population with exact tests using Markov chain methods in GENEPOP 3.4 [30]. Corrections for multiple testing were performed using the false discovery rate (FDR) approach. Genetic differentiation between sampling sites was quantified by computing pairwise estimators of *F*_ST_ according to [31] and their significance was tested by 1000 permutations in GENETIX 4.03 [32]. For all analyses based on allele frequencies in populations (like *F*_ST_), two genotyped individuals from captive population GRZ were removed.

An individual-based Bayesian clustering procedure, implemented in STRUCTURE 2.3.3 [33], was used for individual-based assignments to distinct genetic clusters. The Bayesian model assumes *K* (unknown) populations with different allele frequencies at a set of independent loci. The program was run with 20 independent simulations for each of *K* from 1 to 15, each of 10^6^ iterations, following a burn-in period of 10^5^ iterations. In all simulations an admixture ancestry model and correlated allele frequency model (with λ = 1) was used. The likelihood of *K*; i.e. Ln Pr(*X*|*K*), was used to infer the best number of real populations in the datasets using the method of Evanno *et al*. [34]. We also forced the assignments of individuals to clusters beyond the number considered to maximize the posterior probability of the data. This approach was used to reconstruct the hierarchical relationships among populations, as well as to distinguish between historical processes that are likely to shape the structure (e.g. [35]). The results of 20 replicate runs for each value of *K* were combined using the Greedy algorithm of CLUMPP 1.1.1 [36] and summary outputs for each value of *K* were displayed graphically using DISTRUCT v. 1.1 [37].

• Spatial genetics - microsatellite data

An individual-based spatial approach of Bayesian clustering was used to analyze the spatial structure of the genetic data. We employed the methods implemented in BAPS version 4.1 [38]. BAPS estimates the hidden population substructure by clustering individuals into genetically distinguishable groups based on allele frequencies and linkage disequilibrium. A major advantage compared to most other methods is that the number of populations is treated as an unknown parameter that can be estimated from the dataset. We performed 20 independent runs examining the spatial clustering of groups of individuals (collected at the same locality). Based on the initial results, we tested specific hypotheses by repeating the analyses for a fixed number of populations (i.e. fixed-*K* spatial clustering of groups of individuals).

Isolation by distance was analysed by regressing pairwise estimates of *F*_ST_/(1- *F*_ST_) against ln-distance between sample sites [39]. Mantel tests were used to test the correlation between matrices of genetic differentiation and Euclidean distances between sampling sites by 1000 permutations in GENEPOP. This test was performed for the complete dataset (excluding only two individuals from captive population GRZ) and separately for the three groups of populations defined by preliminary analyses of mtDNA and microsatellite data (i.e. Chefu, LimpN, LimpS; Pop406 was excluded, because it concerns the secondary contact of two differentiated groups - see below; see Table 1 for assignment of populations to particular groups).

To test particular hypotheses of the genetic connectivity among populations, we used the maximum likelihood approach to estimate the effective population numbers and immigration rates as implemented in Migrate v. 3.5.1 [40]. We performed the Likelihood ratio test (LRT) to test the fit of a priori defined scenarios of the gene flow against the full model, assuming *n*-island migration model. In particular, we considered two migration models. First, we assumed that migration only occurs between neighbouring populations along the axis following the course of the main rivers (stepping-stone migration model). Second, we assumed that migration only occurs downstream, i.e. from a higher altitude sites to the lower altitude sites (mediated by floods).

Due to high number of sampling sites in the Chefu region, which would make the model computationally too complex, we performed the Migrate analysis only in the LimpS and LimpN clades (Table 1, Figure 1). We were forced to further omit several sites due to their admixture (Pop406; see below) and position outside the main river axis, given that they impeded the definition of migration scenarios and increased the number of degrees of freedom of the model. Hence, the migration matrix was only evaluated for populations 207, 120, 119 and 326 in the LimpS clade and 13, 8, 2, 1, 50 and 55 in the LimpN clade; the rank representing upstream to downstream linear transects. Migrate was initially run with 10 short and 4 long chains of default settings. Subsequently, the results were used as starting values for extended runs with 10 short chains of 1000 sampled genealogies with 100 increment and 4 long chains of chains of 10000 recorded steps and 100 increment value and a mixing scheme with temperatures ranging from boiling to cool (106, 3, 1.5 and 1) and swapping interval of 1. We monitored if independent runs provided consistent results and in the final runs, defined migration scenarios were used as alternative hypotheses for the LRTs.

### Intrapopulation genetic variation and historical demography

• Genetic variation and bottleneck - microsatellites

Deviations from Hardy-Weinberg equilibrium (HWE) were tested for each locus and population using the Markov chain method in GENEPOP. The number of alleles (*A*), observed (*H*_O_) and expected (*H*_E_, non-biased estimate) heterozygosities were calculated in GENETIX. Allelic richness (*AR*) corrected for sample size by the rarefaction method (i.e. estimated for a minimum sample size of 11 diploid individuals) were calculated for each population in FSTAT 2.9.3.2 [41].

Excess heterozygosity with respect to that expected at mutation-drift equilibrium for the number of alleles present may indicate a genetic bottleneck [42]. We estimated the deviation of gene diversity from mutation-drift equilibrium at the local scale, using BOTTLENECK 1.2.02 [43]. We used a generalized mutation model in which the change in the number of repeat units forms a geometric random variable, with a variance of the geometric distribution fixed to 0.36, which is likely to be the most appropriate value for microsatellites [44]. This estimate was simulated using 1000 permutations in BOTTLENECK by choosing the two-phase mutation model (TPM), with a variance for TPM equal to 0.36 and a proportion of the stepwise mutation model in TPM equal to 0.7. The significance of deviation from the expected heterozygosity under mutation-drift equilibrium was determined with a one-tailed Wilcoxon signed rank test [45] and the results were corrected for multiple tests using the FDR approach (see above).

• Historical demography based on mtDNA

Demographic analyses assume more or less homogeneous populations. Based on preliminary analyses of microsatellites in STRUCTURE and BAPS as well as the haplotype network of mtDNA and distribution of haplotypes, we defined three genetic groups (= populations; see Table 1 for assignment of individuals into the groups). For analysis of historical demography, we used the reduced dataset of 132 longer mtDNA sequences (815 bp) from individuals collected at 32 localities (Table 1). We also excluded 6 individuals from Pop406 (secondary contact of two differentiated lineages - see below) and a sequence from GRZ (laboratory population). Diversity estimates, i.e. number of polymorphic sites (*Np*), number of haplotypes (*Nh*), haplotype diversity (*Hd*), nucleotide diversity (*Pi*, expressed as percentages, i.e. 0.001 = 0.1%), the average number of nucleotide differences (*k*) and Watterson’s estimate of *θ* (*θ* = 4Ne*μ) were calculated using DnaSP v. 5.10.01 [46].

Demographic histories of the three groups were evaluated using several approaches. We first estimated the neutrality indices Tajima’s *D* and Fu’s *Fs* and Ramos-Onsins and Rozas’s *R2* statistic [47] in DnaSP. They are sensitive to population expansion, contraction and structure and return significantly negative (*D*, *Fs*) or small (*R2*) values in the case of recent population expansion, while population decline and/or structure tend to return positive or high values.

Second, we used the DnaSP and ARLEQUIN 3.11 [48] to reconstruct the histogram of pairwise differences in each clade (mismatch distribution; MD) to test for non-random distribution of coalescence events in the sample. ARLEQUIN was used to test the fit of our datasets to the expectations of the sudden demographic expansion model using the sum of squared deviations between the observed and expected mismatch from 1,000 parametric bootstrap replicates. Under the assumption of the (sudden) demographic expansion model the MD also permits estimation of the time of onset of population expansion τ (τ = 2 t* μ; t = time in years, μ = mutation rate per locus of 815 bp). Parameter τ was estimated with DnaSP using the moment method of Rogers [49] assuming the infinite sites model (IFM) as well as with ARLEQUIN using Schneider & Excoffier’s method [50], which relaxes the IFM assumption. Confidence intervals were obtained by a parametric bootstrap approach based on 1000 replicates performed in ARLEQUIN.

Third, we reconstructed demographic history of the main lineages backward in time using the coalescent-based Bayesian skyline plot (BSP) [51] in BEAST, which infers past population size changes from sequence data [52]. Analyses were run twice for each lineage using the HKY model of sequence evolution, which was an appropriate model within each lineage selected with the jModelTest [53] under the AIC criterion. The MCMC simulations were run with 50 million iterations with a sampling increment of 300 and results were checked for convergence and stationarity of different runs in Tracer 1.4.1 and combined in LogCombiner 1.4.8 module.

### Dating of divergence

To estimate the divergence times of the major phylogroups we reconstructed the time-calibrated tree in BEAST using the dominant (putatively ancestral) haplotypes from each clade and single sequence of closely related species *N. kadleci*, *N. orthonotus* and *N. rachovii*. The tree was rooted with *Aphyosemion herzogi* (GenBank: EU885235.1). An appropriate model of sequence evolution (TrN + I; I = 0.63) was selected with the jModelTest [53] and implemented in BEAST with Jeffreys prior for substitution rates. Divergence times were estimated under a Yule branching process with a constant speciation rate as a tree prior and assuming an uncorrelated lognormal relaxed molecular clock [54]. A normally distributed prior with a mean of 0.007 and standard deviation of 0.0010 was used for the mean mutation rate, since it covered the plausible range of 0.70–0.86% substitutions per site per My, as discussed in [27]. The search started with a UPGMA tree and two independent runs of 3×10^7^ generations were conducted with sampling every 1000th generation. The results were checked for convergence and stationarity using Tracer and combined in LogCombiner after a burn-in of the first 10% of generations. Finally, the molecular clock tree was summarised using the TreeAnnotator 1.4.8 module, using the medians as node heights. Given the lack of an internal calibration point, divergence time estimates are likely overestimates of the real time due to the tendency of τ to artificially antedate recent events [55].

## Results

### Genetic diversity of local populations

The mean frequency of microsatellite null alleles per population was less than 5% for all but one locus. However, even for this locus (Nfu_0140_FLI) the mean frequency of null alleles was low (5.25 ± 5.24%) and we did not consider null alleles as a problem in subsequent population genetic analyses (but see further for their possible effect in tests of the HWE). The microsatellite loci used in our study can be considered to be unlinked. From 1841 results of genotypic linkage disequilibrium tests (i.e. with adequate data), only 50 were significant at *p* < 0.05. Most pairs of loci were significantly linked only in one or two populations. Only two pairs showed significant linkage in four (out of 30) populations (Nfu_0023_FLI - Nfu_0038_FLI and Nfu_0010_FLI - Nfu_0041_FLI), which is consistent with their position in the same linkage group (Additional file 1), i.e. exhibiting an inter-marker distance of 7 and 5 cM in *N. furzeri* GRZ strain [56].

**Figure 2:**
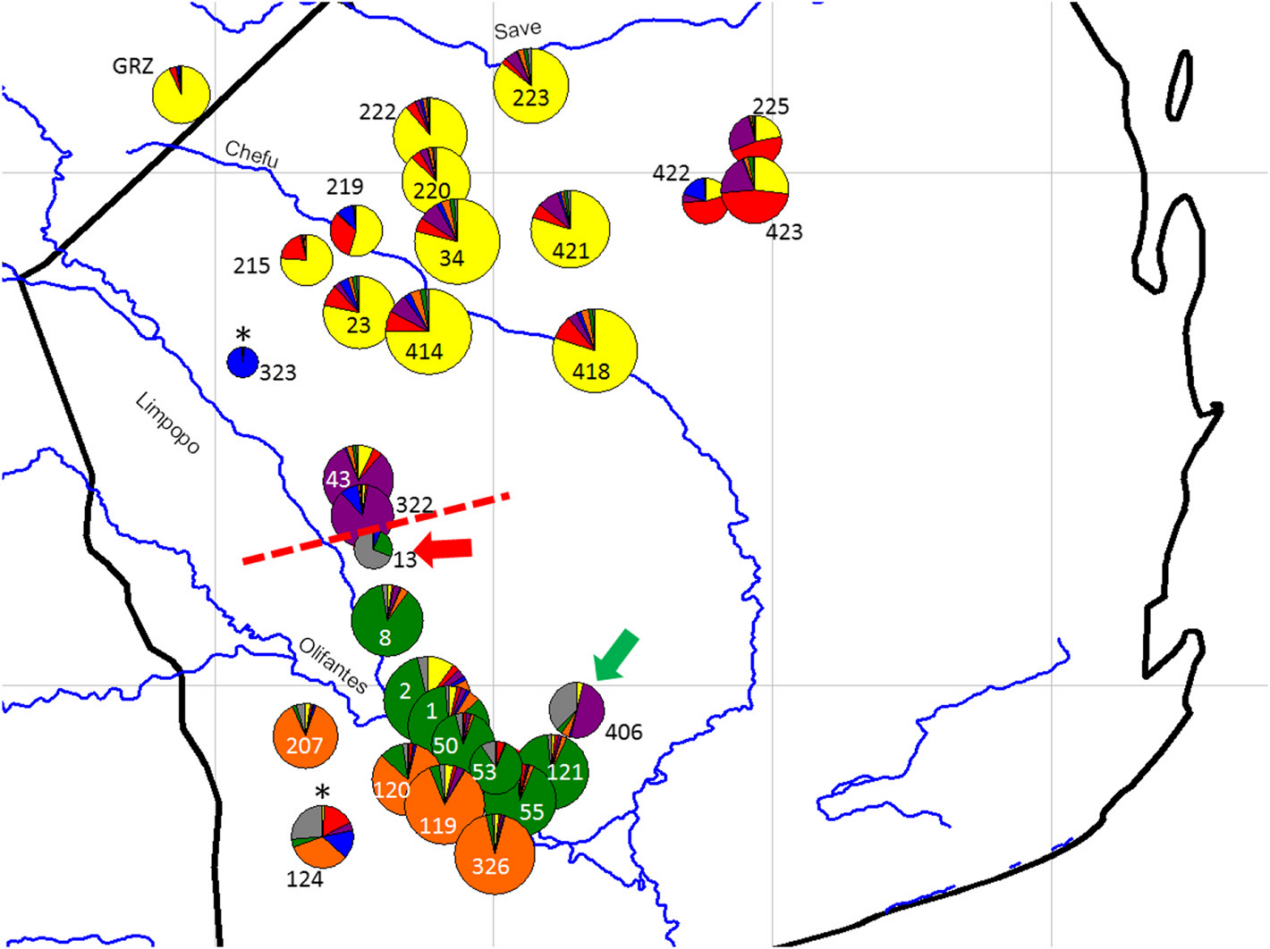
**Genetic variation of nuclear microsatellites assessed using STRUCTURE.** Pie chart colours represent the proportional membership of individuals to microsatellite-based clusters inferred from the model for *K* = 7 (this model is well supported by Evanno et al. approach and provides detailed substructuring; see also Additional file 3). The dashed red line indicates the border between the Chefu and Limpopo haplogroups. The diameter of the pie charts is related to allelic richness (except the captive population GRZ, which is not scaled). Asterisks indicate populations with probable inbreeding (deficit of heterozygotes at four loci; Pop323 and Pop124); the red arrow indicates a population showing a significant bottleneck (Pop13); green arrow indicates secondary contact of two mtDNA lineages (Pop406).

Most populations (25 out of 30) showed deviance from HWE, when calculated over all loci. In most cases deviations from HWE were caused by null alleles present at an increased frequency at some loci and populations. The mean frequency of null alleles for 21 locus-population tests significant after FDR correction (from 13 × 30 = 390 locus-population pairs) was 13.1 ± 5.45% (N = 21), while for the whole dataset the mean frequency of null alleles was only 2.43 ± 4.14% (N = 390). In most populations, the HW disequilibrium was caused by just one or two loci. The only exceptions were peripheral populations Pop124 and Pop323, which showed a deficit of heterozygotes at four loci, suggesting an increased level of inbreeding (Figure 2).

All measures of intrapopulation genetic variation (*H*_O_, *H*_E_, *AR*; Table 2) were strongly correlated (all R > 0.8, p < 0.05) and we describe only the distribution of *AR*. The range of *AR* was 4.85-13.50 (rarefaction estimate for the lowest sample size N = 11). The most variable populations were usually located in the centres of the distribution of the three main genetic groups. The lowest estimates of *AR* were encountered in sites at the periphery of their distribution (Figure 2, Table 2). These local populations (e.g. Pop13, Pop323, and Pop422) are likely to represent recent founder effects, supported by the results of Bottleneck tests, where the only population with a significant heterozygosity excess (after FDR correction) was Pop13, indicating recent decrease in effective population size (Table 2). The diversity of mtDNA at these localities was also low with the only haplotype at Pop13 (n = 3) and two haplotypes differing by 1 bp substitution at Pop323 (n = 7) and Pop422 (n = 3).

**Table 2:** Intrapopulation genetic variability measures on nuclear microsatellites

**Population**	***H***_**E**_	***H***_**O**_	***AR***	***Bottleneck***
1	0.923	0.860	12.599	0.344
2	0.944	0.919	13.500	0.142
8	0.886	0.845	11.097	0.433
13	0.804	0.759	6.142	0.001*
23	0.915	0.877	11.204	0.473
34	0.942	0.923	13.201	0.229
43	0.858	0.796	10.900	0.706
50	0.876	0.860	9.852	0.433
53	0.834	0.839	8.208	0.433
55	0.904	0.860	11.365	0.168
119	0.899	0.857	12.443	0.235
120	0.866	0.817	11.171	0.468
121	0.917	0.867	11.619	0.140
124	0.806	0.704	9.626	0.706
207	0.853	0.805	10.071	0.706
215	0.857	0.800	8.117	0.142
219	0.831	0.820	8.151	0.433
220	0.903	0.844	10.607	0.172
222	0.914	0.856	11.478	0.706
223	0.926	0.893	11.646	0.140
225	0.818	0.789	8.193	0.559
322	0.835	0.774	9.675	0.706
323	0.648	0.605	4.850	0.650
326	0.905	0.859	12.389	0.143
406	0.798	0.794	8.656	0.706
414	0.946	0.871	13.318	0.142
418	0.943	0.851	13.109	0.241
421	0.936	0.866	12.702	0.337
422	0.788	0.721	7.317	0.706
423	0.886	0.866	10.448	0.531

Expected (*H*_E_) and observed (*H*_O_) heterozygosity and allelic richness corrected for sample size (*AR*). *Bottleneck* indicates the probability of mutation-drift equilibrium evaluated by Wilcoxon test in the program Bottleneck (* significant at p < 0.05 after FDR correction).

### Spatial genetic structure over the entire distribution range of the species

**Figure 3:**
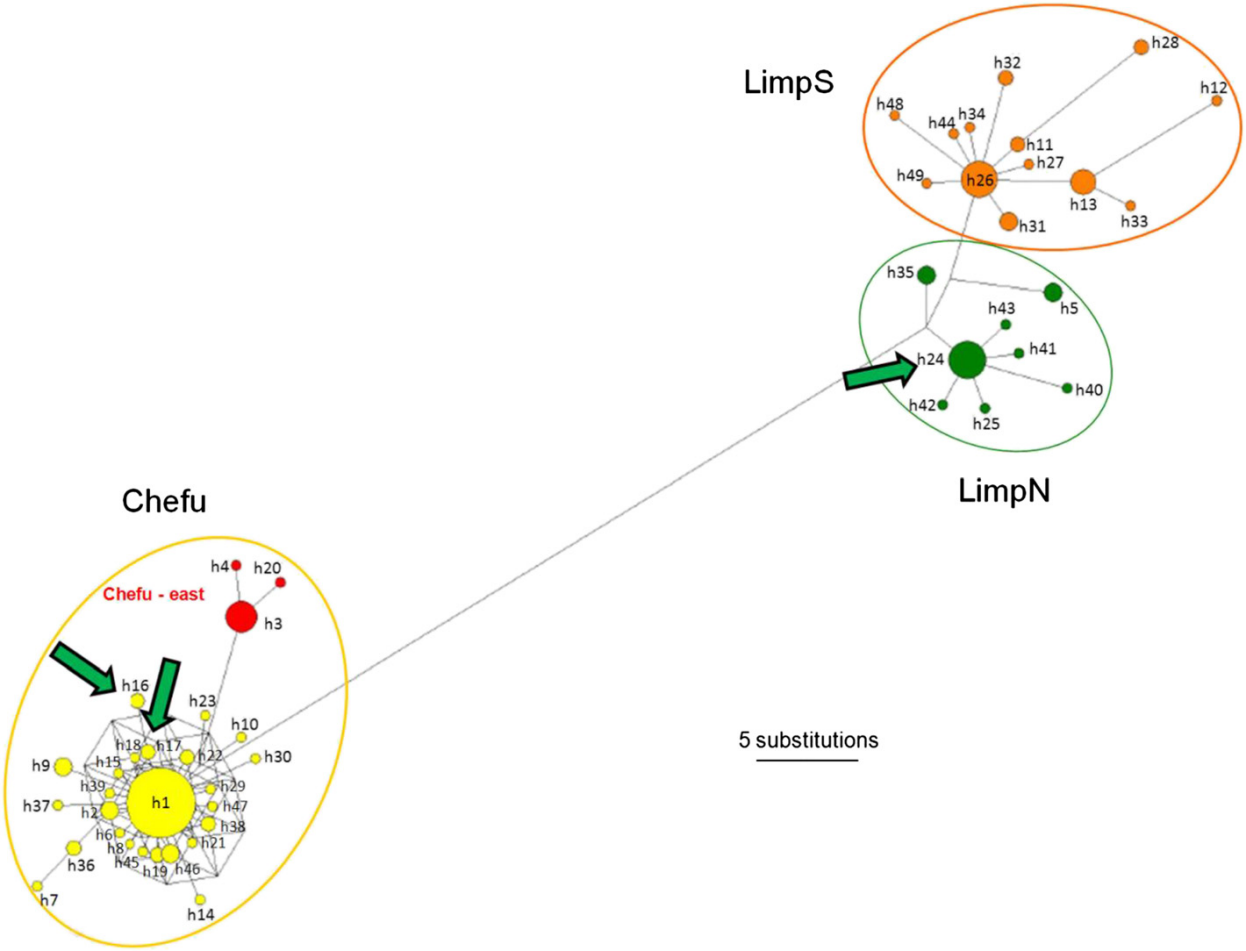
**Median-joining network of 149 sequences of 687 bp of *CYTB *from 36 populations.** Length of branches is proportional to the number of substitutions along a given branch, and circle size is proportional to haplotype frequency. Haplotypes from three main genetic groups (LimpS, LimpN, Chefu) are enclosed by circles of different colours. Private haplotypes from the easternmost localities in the Chefu basin (Pop225 and Pop423) are in red. Green arrows indicate haplotypes co-occurring in Pop406 (haplotypes h16, h17, and h24).

Median-joining network analyses of 149 *CYTB* sequences (687 bp) revealed 49 different haplotypes from two main genetic groups, likely associated with discrete river basins (Figures 1 and 3). The first, northern group was found to inhabit savannah pools across the basin of the intermittent River Chefu. Within this haplogroup, three haplotypes (h3, h4, h20) formed a separate lineage, distributed in the easternmost region of the *N. furzeri* distribution (Figure 3). The second, southern group was clearly subdivided into northern (LimpN) and southern (LimpS) subgroups, with the River Limpopo representing possible barrier. The LimpS group contains pools located within the basins of the Limpopo and Incomati rivers. Population 406 likely represents an example of secondary contact between the Chefu and LimpN mtDNA haplogroups. From six individuals, two had haplotype h24, which is frequent in LimpN populations. However, four individuals expressed private haplotypes for this population, h16 and h17, which belong to the Chefu haplogroup (Figure 3). Sequencing a shorter fragment (481 bp) of *CYTB* in nine additional individuals (not included in any other analyses) revealed the same proportion of Chefu and LimpN haplotypes; six individuals possessed Chefu haplotypes and three individuals the LimpN haplotypes.

Based on microsatellite data, *N. furzeri* in the study area is genetically structured (mean pairwise *F*_ST_ = 0.077 ± 0.0451). Only five of 420 pairwise *F*_ST_ were not significant (Additional file 2) and these five pairs were all geographically close to each other in the centres of their respective haplogroup ranges. On the other hand, the three closest populations (Pop53, Pop55, and Pop121 - all of them belonging to LimpN, with a distance from each other only 1.3-2.7 km) were significantly different, with *F*_ST_ values from 0.015 to 0.045. There were even extremely high pairwise *F*_ST_ between neighbouring populations (e.g. Pop215 and Pop323, 48 km distance within the Chefu group: *F*_ST_ = 0.210) suggesting important barriers to gene flow over short distances and/or strong effect of genetic drift.

The most supported division in Bayesian clustering in STRUCTURE was for *K* = 2, but suitable models according to Δ*K*[34] are also represented by *K* = 5, 7, 9, and 13 (Additional file 3). In the model for *K* = 2, the clustering of populations was congruent with the two main mtDNA haplogroups (i.e. Chefu vs. Limpopo) and, similarly, for *K* = 3 the populations around the River Limpopo roughly diverged to LimpS and LimpN (Additional file 3). Further increases in the number of presumed clusters led to separation of other populations, most often in peripheral parts of the distribution of the three main genetic groups (see Figure 2 for the situation in *K* = 7, where the most distant populations in specific groups were assigned to separate clusters). Population 406, with individuals belonging to the two mtDNA haplogroups (Chefu and LimpN), is genetically homogenous on nuclear microsatellites (Additional file 3) suggesting that there is no reproductive isolation between the two mtDNA lineages.

A similar pattern of genetic structure was observed in the spatial analysis in BAPS (Additional file 3). The best model identified 13 genetic populations, which are almost identical to those in STRUCTURE for *K* = 13 (Additional file 3). The main divergence is between northern (Chefu) and southern (Limpopo) populations (in model *K* = 2). Further increase in the number of clusters led to separation of peripheral populations in all three main groups and separation of left and right banks of the River Limpopo (LimpN and LimpS) (Additional file 3).

The genetic distance between populations was correlated with their geographic distance, when all populations were analysed together (Mantel test, 1000 permutations, p < 0.001). When populations were separated into the three main genetic groups, the IBD pattern remained significant only in the Chefu populations (*p* = 0.001), despite the same trend observed in both LimpN (*p* = 0.062) and LimpS (*p* = 0.085). This pattern was strongly influenced by the genetic distinctiveness of peripheral populations in all three groups, mainly populations 323, 13, and 124 in Chefu, LimpN, and LimpS, respectively (Additional file 2).

The analysis of genetic connectivity for LimpS and LimpN clades in Migrate is summarized in Table 3. All alternative hypotheses always provided significantly lower fit than the full model (LRT tests; *p* < 0.001). This demonstrates that genetic connectivity in both clades strongly deviate from a simple stepping-stone migration model and from a model assuming strictly downstream migration. A further support comes from the fact that the full models indicated that all localities received non-negligible immigration rates from non-neighbouring sites and that several localities in both clades received considerably higher immigration rates from sites located at lower altitude than from any of the upstream sites (Table 3).

**Table 3:** Estimates of population sizes and migration rates obtained from final Migrate runs

(a) LimpS						
	From Pop207	From Pop120	From Pop119	From Pop326		
Pop207 (136 m)	**25.7**	2.2^1^	1.6^1,2^	1.9^1,2^		
Pop120 (56 m)	4.8	**7.4**	7.8^1^	4^1,2^		
Pop119 (48 m)	7.3^2^	6	**4.0**	10.4^1^		
Pop326 (30 m)	2.6^2^	7.9^2^	6.3	**6.7**		
(b) LimpN:						
	From Pop13	From Pop8	From Pop2	From Pop1	From Pop50	From Pop55
Pop13 (129 m)	**0.8**	11.8^1^	8.5^1,2^	10.1^1,2^	5.6^1,2^	13.2^1,2^
Pop8 (118 m)	8.1	**0.9**	5.5^1^	15.2^1,2^	5.6^1,2^	7.0^1,2^
Pop2 (77 m)	10.8^2^	4.4	**1.4**	9.9^1^	5.6^1,2^	10.9^1,2^
Pop1 (59 m)	13.0^2^	4.2^2^	13.1	**1.8**	11.7^1^	5.5^1,2^
Pop50 (49 m)	2.9^2^	11.7^2^	3.4^2^	10.4	**1.6**	9.0^1^
Pop55 (33 m)	11.2^2^	6.1^2^	7.2^2^	5.8^2^	7.9	**2.4**

(a) LimpS clade; (b) Limp N clade. Numbers in bold along the diagonals indicate *θ* (=4Ne*μ) estimates, while other numbers indicate the estimates of immigration rates from respective sites (*M*=*m*/*μ*). Upper indices relate to the alternative hypotheses tested through the LRT. ^1^ indicates those parameters, which were constrained to zero in the model assuming only downward migration, while ^2^ indicates those constrained to zero in the stepping-stone model. Elevation of particular localities is shown in parentheses.

### Historical demography based on mtDNA

**Figure 4:**
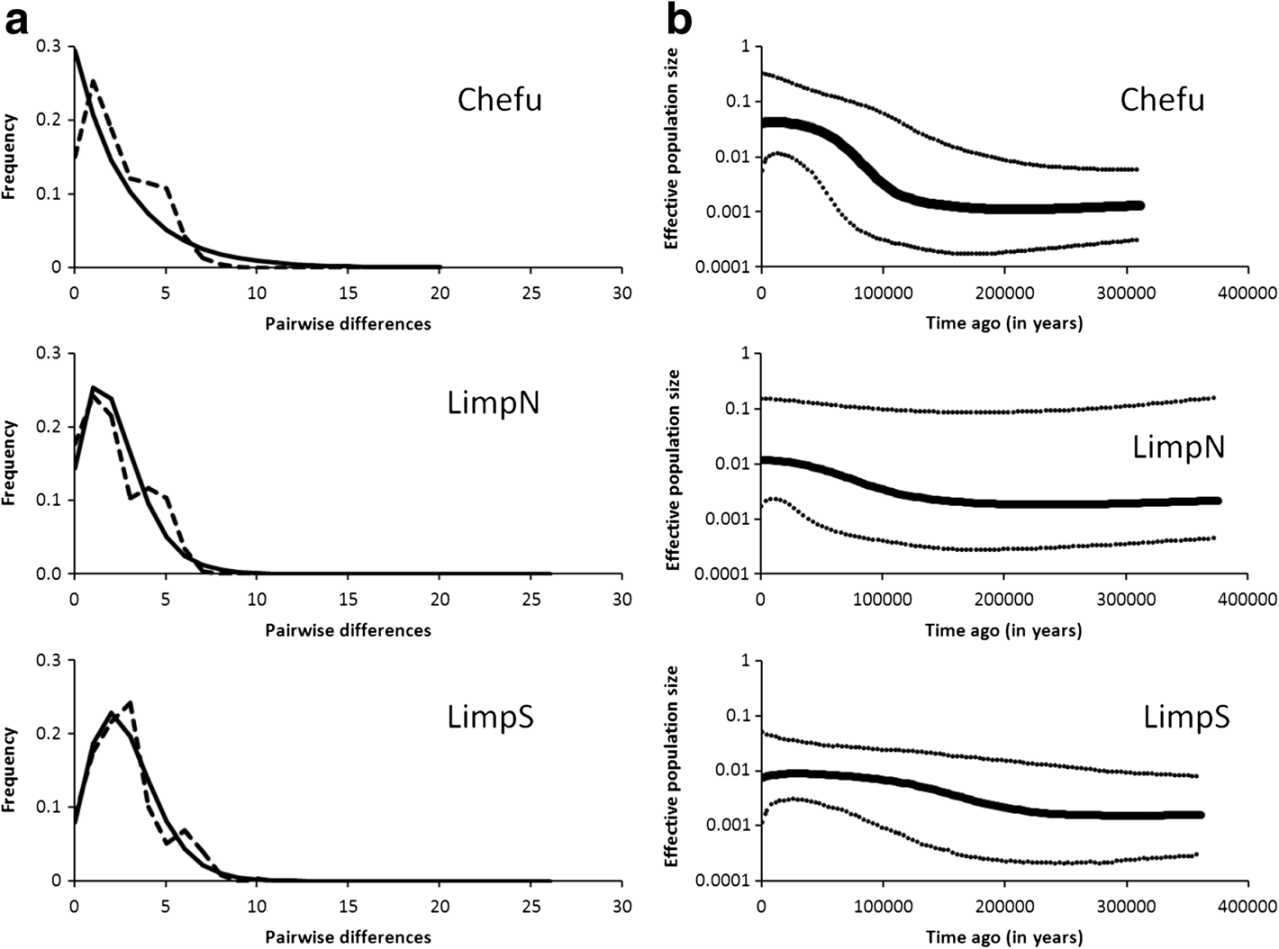
**Mismatch distribution and Bayesian skyline plots for the three mtDNA clades. (a)** Mismatch distribution between mtDNA haplotypes from three major mtDNA clades. Dashed lines connect observed values and solid lines show the expected distribution under a demographic expansion model. **(b)** Bayesian skyline plots for the three main clades, showing changes in effective population size (*N*_*e*_*m*; on a log_10_ scale) over time (in years). The thick solid line depicts the median estimate, while dotted lines represent the 95% highest posterior density intervals.

Comparison of mtDNA variation among the three main genetic groups and the results of neutrality tests are shown in Table 4. All populations significantly fitted the model of sudden growth according to SSD analysis in ARLEQUIN and showed a unimodal pattern (Figure 4a). Bayesian skyline plots indicated moderate (LimpN, LimpS) to intensive (Chefu) population growth in all three genetic groups (Figure 4b). This finding is consistent with neutrality tests that showed a non-significant signal for population growth in the LimpS and LimpN clades (where only the *R2* values were significant), while all tests indicated highly significant deviations from neutrality in the Chefu clade (Table 4). The τ values from DnaSP suggested more recent population expansions compared to estimates by ARLEQUIN, which conforms to a previously reported tendency of the moment method to underestimate the true values [49]. Nonetheless the DnaSP estimates were always included within the 90% CI of ARLEQUIN (Table 4). ARLEQUIN analysis was congruent with the BSP analysis and indicated a more recent population expansion in the Chefu group (mean estimates 70–118 kya according to the method used) compared to two Limpopo groups (112–255 kya) (Table 4). This timing is comparable to that suggested by the BSP analysis (Figure 4b).

**Table 4:** Variation of three main mtDNA haplogroups and analysis of historical demography

**Group**	***N***	***S***	***H***	***Hd***	***Pi (%)***	***k***	***θ***	***Tajima’s D***	**Fu’s *Fs***	***R2***	**τ Arlequin *(5% qt-mean-95% qt)***	**τ DnaSP**
LimpN	22	13	10	0.823 ± 0.073	0.271 ± 0.052	2.212	0.00438	−1.34485	−3.72	0.0741*	0.584–2.904–4.283 (255 ky)	1.275 (112 ky)
LimpS	31	20	13	0.912 ± 0.026	0.345 ± 0.049	2.813	0.00614	−1.51702	−4.477*	0.0654*	1.232–2.789–3.882 (244 ky)	1.873 (164 ky)
Chefu	79	33	27	0.849 ± 0.030	0.294 ± 0.030	2.393	0.0082	−2.02665*	−20.337*	0.0330**	0.006–0.799–5.289 (70 ky)	1.350 (118 ky)
All groups	132		50									

These analyses are based on 815 bp of *CYTB. N* number of individuals, *S* number of segregating sites, *H* number of haplotypes, *Hd* haplotype (gene) diversity ± SD, *Pi* nucleotide diversity (in%), *k* average number of nucleotide differences, *θ* Watterson’s estimate of theta (per site) from S, *τ* the parameter of onset of population expansion assuming the stepwise growth model (τ = 2 t*MU; t = time in years, MU = mutation rate per locus). Fu’s *Fs* significance p < 0.01 is marked by *, Tajima’s *D* significance p < 0.05 is marked by *, *R2* significance p < 0.05 is marked by *, p < 0.01 by **.

### Divergence times among dominant haplotypes in clades

Assuming 0.007 substitutions per site per Mya, the clade LimpS diverged from LimpN approximately 0.99 Mya (0.02-8.39), while the Chefu clade diverged from the Limpopo around 3.64 Mya (0.43-19.95). The age of the whole F clade *sensu*[28] (i.e. *N. furzeri* + sister species *N. kadleci*) was estimated at around 6.72 Mya (1.58 – 31.99). Note that the estimated divergence time likely represents the upper limits of the actual timing [55].

## Discussion

We demonstrated a deep and ancient division of *N. furzeri* populations into two major clades, likely associated with river basins (northern: Chefu and southern: Limpopo + Incomati). Finer subdivision of the southern clade is facilitated by the River Limpopo, which apparently serves as a barrier to gene flow between the two subclades. Within clades, high genetic structuring of populations inhabiting individual pools likely stems from limited dispersal between pools and frequent founder effects and bottlenecks. Significant isolation by distance within main genetic groups suggests that dispersal occurs in small steps. Genetic connectivity analysis further demonstrated frequent migration to the higher altitude, discounting hypothesis that dispersal is strictly related to major floods associated with river routes. Demographic analyses revealed ongoing expansion of the main clades from their putative refugia.

### Historical processes and evidence for two savannah refugia

The existence of two major clades of *N. furzeri* was unambiguously confirmed by both mitochondrial and nuclear markers. This division suggests a historical split of *N. furzeri* populations between two refugia. Based on current genetic diversity at microsatellite markers and haplotype distribution, the first refugium was probably located in the central Chefu basin. The second refugium is hypothesised to have been located in the left bank of the Limpopo, in the region between the confluences of the Limpopo with the Rivers Olifantes and Chefu (Figure 2). The estimated divergence time of 3.6 Mya places the divergence event within a period during the Pliocene characterised by a relatively warm and humid climate [57]. Such climatic conditions may have resulted in fragmentation of savannah biome into discrete units separated by widely distributed forests and forested areas may have played an important role in splitting the range of *N. furzeri* into two refugia. Populations of *N. furzeri* may have expanded during continuous aridification of the region at the end of Pliocene and during cold and dry periods of Pleistocene, when the open savanna conditions had become more prevalent [58]. In one of these dry periods, the fish from the southern refugium probably colonized the right bank of the River Limpopo, including the pools belonging to Incomati River basin. Interestingly, there are no apparent morphological differences between the isolated clades, except for the relative proportion of male colour morphs [19], though a rigorous comparative morphological study is lacking.

The River Chefu (sometimes referred to as the River Changane in its lower course) is at present a relatively small river, with a large portion of the river bed dry for much of the year, including an extensive period of the rainy season (notably in its upper and middle reaches). The area forms a large pan confined by hills in the south-west and the River Save basin in the north (Figure 1). To the east the catchment is separated from the Indian Ocean by a series of parallel ridges, effectively forming an enclosed basin and indicating a potential existence of a large lake in mesic periods (Figure 1). While pedological and palynological data are lacking, the existence of grassland (rather than woodland) savannah, flat topography, sandy soils and numerous temporary lakes in that area lead us to speculate that an extensive region of the south-eastern part of the Chefu basin may have been continuously flooded during wetter climatic periods and formed an effective barrier to *N. furzeri* dispersal.

The distribution of genetic variation suggests further splitting of southern lineage at an estimated time of 1 Mya. Dating of this event corresponds with the last wet period in the African climate record (dated as 1.1-0.9 Mya), followed by a further aridification in late Pleistocene [9]. Well developed riverine forests along Limpopo River may have functioned as an efficient barrier to gene flow for savannah-dwelling animals, as documented for rodents of the genus *Aethomys*[59]. Thus, unusually for a fish, a large river (the Limpopo) has formed a barrier to gene flow between populations. Rivers, including their margins and active alluvium, represent an inhospitable environment for *Nothobranchius* and dispersal across the main channel may be an extremely rare event. An analogous pattern has been described for riverine cichlid fish from the lower Congo basin, where powerful rapids in the main river form a strong barrier to dispersal between shallow-water cichlids species, effectively isolating populations on opposite banks of the river [60].

We identified a single population with secondary contact between the Chefu and Limpopo groups (Figure 1), where mtDNA haplotypes from both haplogroups were unambiguously identified, while nuclear markers revealed no intrapopulation genetic structure (Additional file 3), suggesting a lack of reproductive isolation between the two mtDNA clades. Interestingly, our transect sampling at the other potential contact zone between the two major groups identified a region where group-specific populations occur only 11 km apart (Pop325 and Pop13; Figure 1), with no evidence of secondary contact despite intensive sampling in that region and no clear geographical or geological barrier. Whether this region is only an arbitrary location where distribution of the two clades is bordering or whether it represents putative suture zone (*sensu*[61]) needs to be confirmed by studies of other taxa. Suture zones, where clades from separate refugia meet, are congruent among taxa in Europe and North America [2,62] and have also recently been confirmed for savannah-dwelling mammals in Africa, separating eastern and southern savannah regions [4]. It is notable that despite a putative Pliocene divergence (3.6 Mya), there is no reproductive barrier between the two clades, as indicated by microsatellite data for Pop406. In contrast, similarly old divergence events have led to speciation in several taxa in the Northern Hemisphere [2], murid rodents of Sudanian savannah in western Africa (e.g. [63,64]), and intralacustrine radiations of cichlid fishes in African lakes [65]).

### Demographic analysis: contemporary processes

Our data indicate that individual *N. furzeri* populations are subject to strong fluctuations in size, extinctions, and recolonizations, suggesting a strong metapopulation structure over an evolutionary time scale. These conclusions are based on evidence for recent genetic bottlenecks, low allelic richness and Hardy-Weinberg disequilibria, particularly in populations at the periphery of the range. Strong geographical structuring, with significant *F*_ST_ values, even between adjacent populations that are separated by 1.5 km of flat woodland savannah habitat, suggests that dispersal between pools is a rare event on an ecological time scale.

Dispersal of *N. furzeri* populations across savannah is slow and likely to be accomplished through longer-term geological events at the larger scale. Propagules of a large number of aquatic invertebrates and macrophytes have been reported to be effectively dispersed by large herbivores [24] or water birds, though a hypothesis for the animal-mediated dispersal of *Nothobranchius* eggs encased in mud over longer distances appears unlikely given the limited contact between the clades and populations. Similarly, dispersal aided by waterbirds would result in weaker geographical structuring. On the other hand, genetic connectivity analysis clearly demonstrated that dispersal is not unidirectional and propagules (eggs or adults) from the lower altitude sites sometimes colonize higher altitude pools. This may indicate animal-mediated dispersal on short distances. At present, these ambiguous outcomes make *Nothobranchius* dispersal mode elusive, but a further insight may be provided by ongoing analysis of sympatric congeners.

The Chefu group, with strong isolation by distance, a star-like haplotype network, and decreasing genetic diversity from the centre to the periphery of its range has all the attributes of an expanding population, supported by analysis of its historical demography. This expansion is dated to begin approximately 160–250 kya, corresponding to a very dry phase in the African climate (penultimate glacial/interpluvial), and predating a series of extreme droughts reported from eastern Africa [10], which resulted in the disappearance of several large lakes such as Lake Victoria [66]. Weaker, but comparable, signals of an expansion indicate a similar pattern for the two Limpopo groups.

### Consequences of genetic structure for ageing research

*Nothobranchius furzeri* represents an emerging model for ageing research and different captive strains of *N*. *furzeri* of different geographic origin show large differences in captive lifespan and senescence rates [67]. Our study confirms that populations of *N. furzeri* are strongly structured and suggests that two large groups represent ancient clades. This finding contrasts with other fish genomic models (three-spined stickleback, cichlid lake radioations) [65,68] which are genetically less diversified despite their greater morphological variability. The current range of *N. furzeri* spans a strong cline in environmental conditions. More arid conditions are found in the Chefu river basin where savannah pools are filled with water for a shorter period, while relatively more mesic conditions are found in the southern part of its range [18,67]. We demonstrated that both main *N. furzeri* clades have ranges encompassing a substantial variation in environmental conditions, making comparative studies possible at two levels of divergence. As genome sequencing of *N. furzeri* is underway [69] and genomic regions controlling simple traits have been mapped by linkage analysis [70], an intriguing possibility includes comparative genomics aiming at identifying genetic variation associated with different lifespans [56,68].

### Availability of supporting data

Sequences of CYTB of 149 individuals of *N. furzeri* were submitted to GenBank (Accession numbers KC777039-KC777187), and microsatellite dataset was deposited to the database DRYAD (doi:10.5061/dryad.30sq1).

## Conclusions

In this study we provide the first detailed analysis of the phylogeographic patterns and demographic processes in a short-living fish *N. furzeri*, a newly emerging model in aging research. By using the comprehensive sampling covering the entire distribution range of the species and by a combination of mitochondrial and nuclear genetic markers we demonstrated: (1) ancient (pre-Pleistocene) divergence between the two main *N. furzeri* lineages, their recent secondary contact and lack of reproductive isolation; (2) important genetic structuring attributed to the fragmented nature of their environment and isolation-by distance, suggesting that dispersal is limited, occurs over short distances and is not directly associated with river routes; (3) an apparent role of the River Limpopo as a barrier to dispersal and gene flow.

## Competing interests

The authors declare that they have no competing interests.

## Authors’ contributions

MR and JB conceived and designed the study, MR, MP, RB, VB and JB collected samples, VB conducted genotyping, VB, JB and KJ analysed the data, AC and KR contributed primers and samples of the captive GRZ strain, and JB, MR and VB wrote the paper. All authors read and approve the final manuscript.

## Authors’ information

This is a part of master thesis of VB, supervised by JB and MR. They are all interested in evolutionary processes influencing African biodiversity using fish and mammals as model animal groups. MP and RB are working in MR’s lab on the factors affecting life histories of *Nothobranchius* fishes, the research of AC and KR is focused on aging processes using killifishes as one of the models.

## Supplementary Material

Additional file 1 **Protocols for genotyping microsatellites and mtDNA.** (a) Genotyping of microsatellites. (b) Genotyping of mtDNA. Click here for file

Additional file 2 **Pairwise *F***_**ST**_** and isolation by distance.** (a) Matrix of pairwise *F*_ST_ values calculated in Genetix. Highlighted values indicate pairs with non-significant *F*_ST_ values (i.e. p > 0.01). (b) Correlation between Ln(distance) and linearized pairwise *F*_ST_ values (*F*_ST_ /(1- *F*_ST_)) tested with Mantel tests (1000 permutations). Isolation by distance was calculated separately for the three population groups and for the whole dataset. Red points indicate pairwise *F*_ST_ including most isolated localities in all three main groups; i.e. Pop323 in Chefu, Pop13 in LimpN, and Pop124 in LimpS. Click here for file

Additional file 3 **Additions to analysis of population genetic structure.** (a) Evaluation of 20 runs in STRUCTURE for each number of presumable clusters from *K* = 2 to *K* = 15. (i) Likelihood of models in STRUCTURE for increasing number of populations (*K*); (ii) Estimation of the best *K* division according to Evanno *et al*. (2005). The most supported division is for *K* = 2, but suitable models are represented also by *K* = 5, 7, 9, and 13. (b) Assignment of individuals to particular populations using models for *K* = 2 to 15 in STRUCTURE (based on 13 microsatellite loci). Codes for localities correspond to Figures 1 and 2, the names on the left indicate the mtDNA haplogroup. Population 406 with mtDNA from two haplogroups (Limpopo N and Chefu) is marked with a red arrow. (c) Spatial clustering of populations constructed in the program BAPS based on 13 microsatellite loci. Identical colours represent populations with similar genotypic composition. The best model suggests clustering into 13 different populations, therefore indicating strong genetic differentiation among the study populations. We also used suboptimal models to show the hierarchical structure of the sampled populations. Click here for file
