# Supplementary material for: Strong population genetic structuring in an annual fish, Nothobranchius furzeri, suggests multiple savannah refugia in southern Mozambique
Source: BMC Evol Biol. 2013 Sep 12;13:196. doi: 10.1186/1471-2148-13-196 (PMC4231482; doi:10.1186/1471-2148-13-196)
Supplement: Additional file 1 — Protocols for genotyping microsatellites and mtDNA. (a) Genotyping of microsatellites. (b) Genotyping of mtDNA. [file 1471-2148-13-196-S1.doc]

**Additional file 1** Protocols for genotyping of microsatellites and mtDNA

***(a) Genotyping of microsatellites***

Microsatellite markers were developed previously using the *N. furzeri* genomic sequence (~10 Mb, for details on genomic sequencing see Reichwald *et al*., 2009). Microsatellites comprising 1-5 nucleotides were identified using the program Sputnik (http://espressosoftware.com/sputnik) and parameters –v 1 –u 5. Microsatellite flanking primers were designed in genomic sequences with: (i) only one microsatellite, (ii) at least 20 repeat units for dinucleotide repeats or at least 10 repeat units for tri- and tetranucleotide repeats, (iii) at least 100 nt of flanking sequences, (iv) perfect repeats and (v) mononucleotide stretches were excluded. Primers were tested in one *N. furzeri* GRZ specimen and amplicons sequenced and analyzed as described (see Dataset S4 in Kirschner *et al*., 2012) to confirm the presence and number of repeat units of a microsatellite. Primers were subsequently tested in other *N. furzeri* populations and employed here for genotyping if amplification and genotyping by PCR was successful across populations.

Table 1: Experimental analysis of microsatellites

| Marker-ID | Label | µM | Set | A | Range (bp) |
| --- | --- | --- | --- | --- | --- |
| **Nfu_0006_FLI** | FAM | 0.3 | 1 | 55 | **195-355** |
| **Nfu_0009_FLI** | VIC | 0.3 | 1 | 47 | **140-260** |
| **Nfu_0010_FLI** | NED | 0.1 | 1 | 38 | **165-245** |
| **Nfu_0012_FLI** | PET | 0.075 | 1 | 35 | **145-245** |
| **Nfu_0016_FLI** | VIC | 0.2 | 3 | 56 | **175-300** |
| **Nfu_0020_FLI** | NED | 0.5 | 4 | 66 | **180-310** |
| **Nfu_0023_FLI** | FAM | 0.2 | 4 | 56 | **215-235** |
| **Nfu_0027_FLI** | VIC | 0.15 | 2 | 71 | **155-305** |
| **Nfu_0029_FLI** | PET | 0.1 | 2 | 45 | **175-285** |
| **Nfu_0030_FLI** | FAM | 0.1 | 2 | 54 | **150-265** |
| **Nfu_0038_FLI** | NED | 0.15 | 3 | 90 | **190-375** |
| **Nfu_0041_FLI** | VIC | 0.2 | 4 | 55 | **175-280** |
| **Nfu_0140_FLI** | NED | 0.2 | 2 | 75 | **165-355** |

*µM*: final concentration of each forward and reverse primer in a multiplex PCR

*Set*: number of multiplex PCR set

*A*: number of alleles

*Range*: length of obtained PCR products across *N. furzeri* populations

**Table 2: Primer sequences and marker features in GRZ strain of *N. furzeri***

| **Marker ID** | **Linkage group**  **in GRZ*** | **[repeat motif] no. of**  **repeat units in GRZ** | **Forward Oligo Sequence** | **Reverse Oligo Sequence** | **PCR product size in GRZ [bp]** |
| --- | --- | --- | --- | --- | --- |
| Nfu_0006_FLI | 12 | [AC]39 | GCAAGCAGCACCCTTTATTTC | GAAAGTAGGGGTCCCACACA | 281 |
| Nfu_0009_FLI | 17 | [GT]23 | GAGCGGGGACAAAGGTTTG | GGAGTTTTCCCCTTTTCAGG | 174 |
| Nfu_0010_FLI | 05 | [AC]19 | GGCTCCTGACAATCCTCCTA | TGAGAGCTGTGACTGATGTGG | 195 |
| Nfu_0012_FLI | 15 | [AC]22 | GATGCCACGCAGATAAAC | CATCTGTGTTTAGGCTGGTC | 185 |
| Nfu_0016_FLI | 20 | [GT]34 | TGCTGGCATCATCACTTCAC | GAAGAATGGCTTGTGGGAGT | 227 |
| Nfu_0020_FLI | 13 | [AC]36 | CTCGTTGTACTTCCAAGAGG | TACCGCTTTGGCTAAACACC | 233 |
| Nfu_0023_FLI | 07 | [GT]27 | GGTTAGGGTTAGGGTGAATC | GTACAGCACCTGCCTTAGTC | 303 |
| Nfu_0027_FLI | 16 | [AC]38 | GGACCACAGAGCAAAAGGAG | AGTTTTTGCCCCACTGTACG | 219 |
| Nfu_0029_FLI | 10 | [AC]18 | GTTTGAAAACCCACAATGCAC | CGCACATCTGATCACTCTCA | 221 |
| Nfu_0030_FLI | 11 | [GT]26 | CAGAAGCTAAAGGCCAGACG | GGGAAACAATAGGGAACCAC | 188 |
| Nfu_0038_FLI | 07 | [AC]26 | CAGTAGGAGGGAGAAGCAG | CTTTGTCAGCTTGCTCTAGG | 227 |
| Nfu_0041_FLI | 05 | [AC]14 | CACACATGGGGTGACTGATT | GACGCAGTGAGTGATTTTATG | 187 |
| Nfu_0140_FLI | 19 | [AC]13 | TGTTTACGCGAGTGATGG | GATGTTCTGATGTGGGTCAG | 187 |

* Linkage group is based on second-generation linkage map of GRZ strain of *N. furzeri* from Kirschner *et al.* (2012)

Temperature profile of PCR for microsatellites:

95°C (15 min) - 1 cycle

94°C (30 s) 56°C (90 s) 72°C (60 s) - 35 cycles

60°C (30 min) - 1 cycle

**Fragment Analysis**

The PCR products (1 µL) were added to a denaturing mixture of size standard (Genescan®, LIZ500, Applied Biosystems) and formamide. After 5 min denaturation at 96°C and 2 min cooling on ice, the mix was run on the ABI Prism® 3130 Genetic Analyzer (Applied Biosystems). DNA fragments were analysed using GeneMapper® v. 3.7 (Applied Biosystems).

***(b) Genotyping of mtDNA***

Primers were designed on the basis of the mtDNA sequence of *N. furzeri* (GenBank Accession NC_011814): FW 40 (GCA AAT GAC TCC CTA ATT GAC C) and REV1019 (CCT CCA ATT CAT GTT AGG GTG).

Temperature profile of PCR for cytochrome *b:*

94°C (2 min) - 1 cycle

94°C (30 sec) 60°C (30 sec) 72°C (3 min) - 30 cycles

72°C (7 min) - 1 cycle

PCR products were sequenced from both sides by the Sanger method using BigDye Terminators v. 3.1 chemistry (Applied Biosystems).

***References cited:***

Kirschner, J., Weber, D., Neuschl, C., Franke, A., Bottger, M., Zielke, L., Powalsky, E., Groth, M., Shagin, D., Petzold, A., Hartmann, N., Englert, C., Brockmann, G. A., Platzer, M., Cellerino, A. & Reichwald, K. (2012) Mapping of quantitative trait loci controlling lifespan in the short-lived fish *Nothobranchius furzeri* - a new vertebrate model for age research. *Aging cell*, **11**, 252-261.

Reichwald, K., Lauber, C., Nanda, I., Kirschner, J., Hartmann, N., Schories, S., Gausmann, U., Taudien, S., Schilhabel, M. B., Szafranski, K., Glöckner, G., Schmid, M., Cellerino, A., Schartl, M., Englert, C. & Platzer, M. (2009) High tandem repeat content in the genome of the short-lived annual fish *Nothobranchius furzeri*: a new vertebrate model for aging research. *Genome Biology*, **10**, R16.
