# Supplementary material for: Strong population genetic structuring in an annual fish, Nothobranchius furzeri, suggests multiple savannah refugia in southern Mozambique
Source: BMC Evol Biol. 2013 Sep 12;13:196. doi: 10.1186/1471-2148-13-196 (PMC4231482; doi:10.1186/1471-2148-13-196)
Supplement: Additional file 3 — Additions to analysis of population genetic structure. (a) Evaluation of 20 runs in STRUCTURE for each number of presumable clusters from K = 2 to K = 15. (i) Likelihood of models in STRUCTURE for increasing number of populations (K); (ii) Estimation of the best K division according to Evanno et al. (2005). The most supported division is for K = 2, but suitable models are represented also by K = 5, 7, 9, and 13. (b) Assignment of individuals to particular populations using models for K = 2 to 15 in STRUCTURE (based on 13 microsatellite loci). Codes for localities correspond to Figures 1 and 2, the names on the left indicate the mtDNA haplogroup. Population 406 with mtDNA from two haplogroups (Limpopo N and Chefu) is marked with a red arrow. (c) Spatial clustering of populations constructed in the program BAPS based on 13 microsatellite loci. Identical colours represent populations with similar genotypic composition. The best model suggests clustering into 13 different populations, therefore indicating strong genetic differentiation among the study populations. We also used suboptimal models to show the hierarchical structure of the sampled populations. [file 1471-2148-13-196-S3.pptx]

## Slide 1
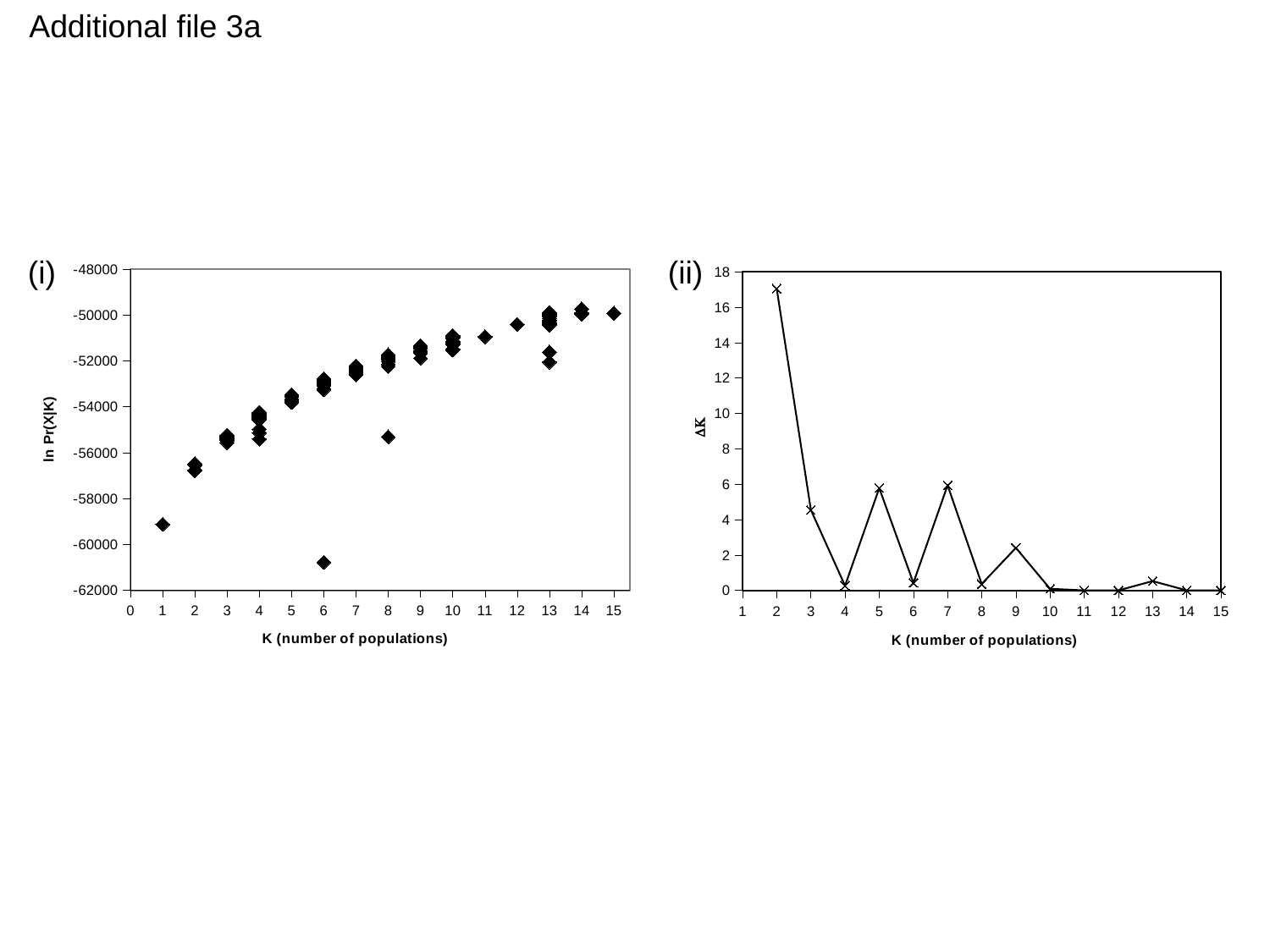

Additional file 3a
### Chart
| Category | |
|---|---|
### Chart
| Category | K ln Pr(X|K) |
|---|---|(i)
(ii)

## Slide 2
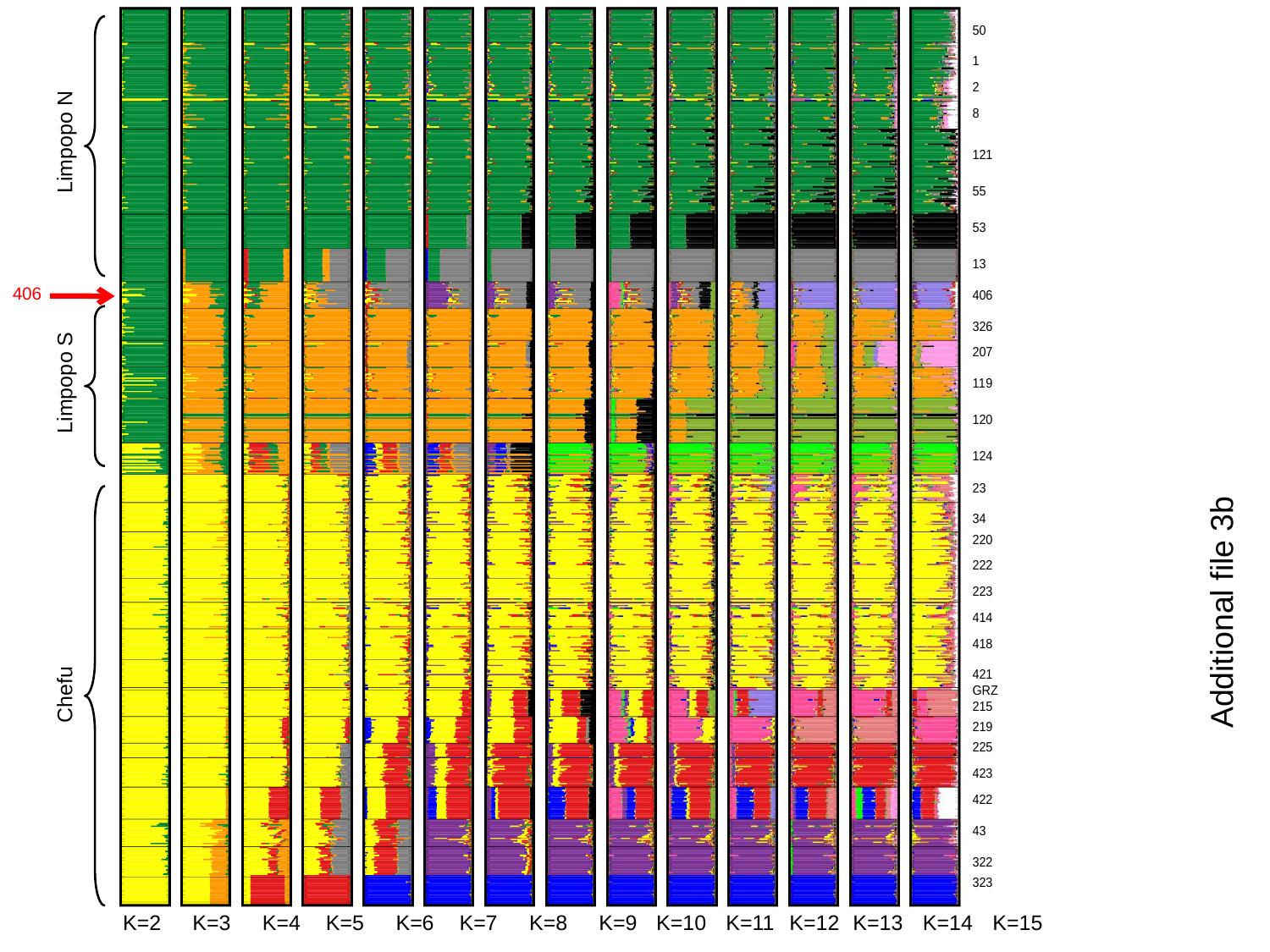

Limpopo N
406
Limpopo S
Additional file 3b
Chefu
K=2
K=3
K=4
K=5
K=6
K=7
K=8
K=9
K=10
K=11
K=12
K=13
K=14
K=15

## Slide 3
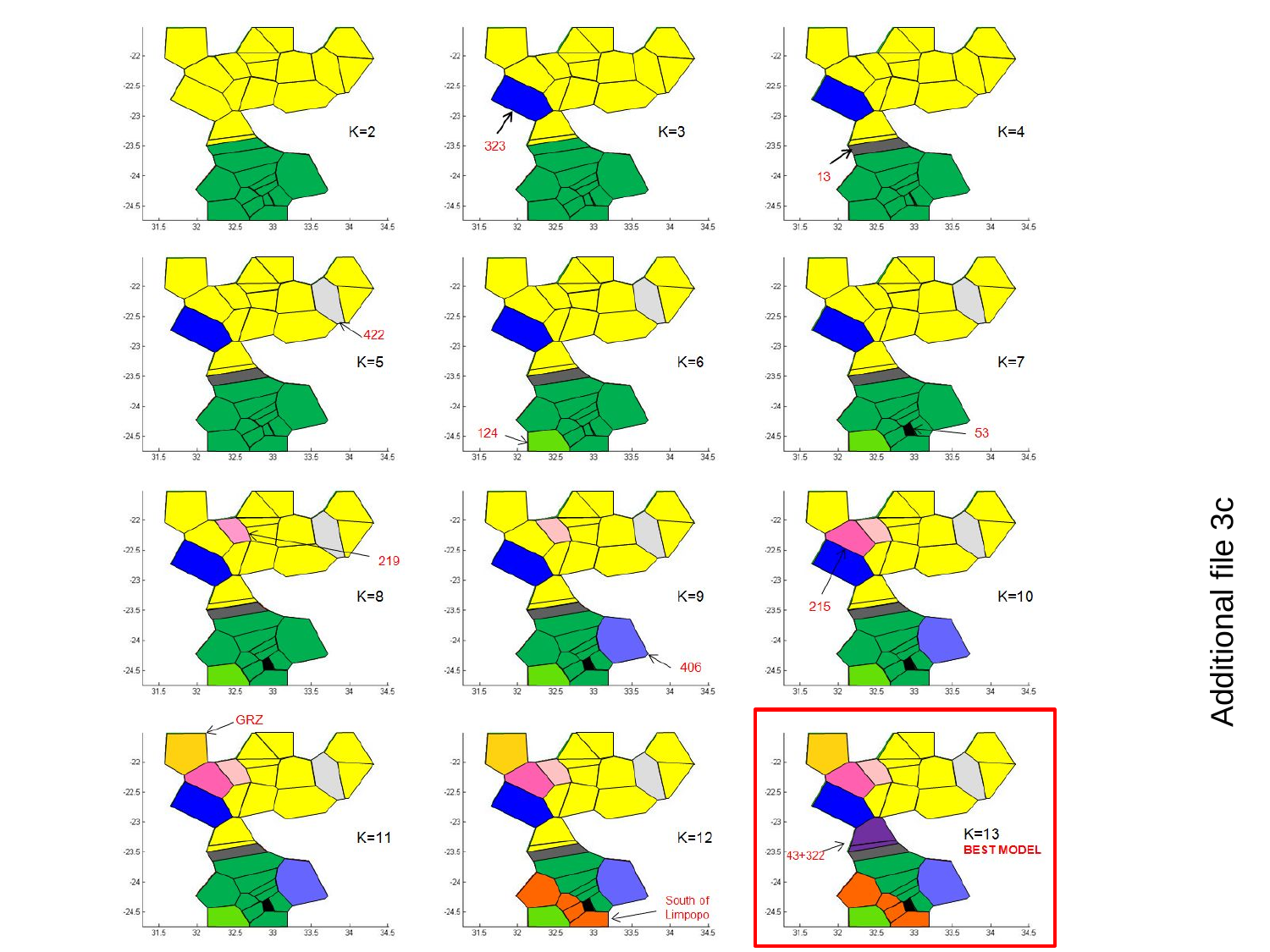

Additional file 3c
